# Loss of lysyl oxidase-like 3 causes cleft palate and spinal deformity in mice

**DOI:** 10.1093/hmg/ddv333

**Published:** 2015-08-24

**Authors:** Jian Zhang, Rui Yang, Ziyi Liu, Congzhe Hou, Wen Zong, Aizhen Zhang, Xiaoyang Sun, Jiangang Gao

**Affiliations:** Institute of Developmental Biology, School of Life Science, Shandong University, 27 Shanda Nanlu, Jinan 250100, China

## Abstract

In mammals, embryonic development are highly regulated morphogenetic processes that are tightly controlled by genetic elements. Failure of any one of these processes can result in embryonic malformation. The lysyl oxidase (LOX) family genes are closely related to human diseases. In this study, we investigated the essential role of lysyl oxidase-like 3 (LOXL3), a member of the LOX family, in embryonic development. Mice lacking LOXL3 exhibited perinatal lethality, and the deletion of the *Loxl3* gene led to impaired development of the palate shelves, abnormalities in the cartilage primordia of the thoracic vertebrae and mild alveolar shrinkage. We found that the obvious decrease of collagen cross-links in palate and spine that was induced by the lack of LOXL3 resulted in cleft palate and spinal deformity. Thus, we provide critical *in vivo* evidence that LOXL3 is indispensable for mouse palatogenesis and vertebral column development. The *Loxl3* gene may be a candidate disease gene resulting in cleft palate and spinal deformity.

## Introduction

Lysyl oxidase (LOX) is an extracellular copper-dependent amine oxidase that has been traditionally believed to catalyse the covalent cross-linking of several fibrillar collagen types and the formation of desmosine and isodesmosine cross-links in elastin. The LOX family has up to five members (LOX and LOX-like proteins 1 through 4), and each member is characterized by a highly conserved amino acid sequence at the C-terminus end that includes the copper binding site, residues for carbonyl co-factor formation, and the cytokine receptor-like domain (1,2). Compared with LOX and LOXL1, there are four scavenger receptor cysteine-rich (SRCR) domains in the N-terminal region of LOXL2, LOXL3 and LOXL4 (1–3). These SRCR domains, which are likely unrelated to catalytic activity, may confer novel distinct biological roles to these amine oxidases.

All five family members are located on different chromosomes in humans and mice (1–9). LOX family members are widely expressed in multiple tissues, and their expression sites are not completely consistent (10), suggesting that every member of the LOX family may have a unique function in different types of tissue. LOX is critical during embryogenesis for the structural stability of the aorta and diaphragm. *Lox*-targeted mice (LOX^−^/^−^) exhibit perinatal lethality, cardiovascular instability with ruptured arterial aneurysms and diaphragmatic rupture (10,11). LOXL1-deficient mice are viable, but they have deposits of abnormal elastic fibres in the uterine tract postpartum, and they develop pelvic organ prolapse, enlarged airspaces in the lung, loose skin and vascular abnormalities with concomitant tropoelastin accumulation (12). LOXL1 serves both as a cross-linking enzyme and as an element of the scaffold to ensure spatially defined deposition of elastin. In humans, LOXL3 and a variant LOXL3-sv1 have high amine oxidase activities in several collagen types and were suggested to have a possible functional role in bone development or maintenance (13). Recently, in a family with autosomal recessive Stickler syndrome, a missense variant (exon12:c.2027G>A) in the human *LOXL3* gene has been identified (14). Stickler syndrome is commonly caused by mutations in different collagen genes, namely COL2A1, COL11A1 and COL11A2 (autosomal dominant inheritance) and COL9A1 and COL9A2 (autosomal recessive inheritance) (15,16). These reports suggest that there is a possible link between *LOXL3* gene and collagen. However, the *in vivo* function of *LOXL3* gene remains unclear as of now. To investigate a possible role for LOXL3, we generated LOXL3-deficient mice (*Loxl3*^−/−^) by a conditional gene targeting strategy (homologous recombination and Cre-recombination). In this study, we discovered that *Loxl3*^−/−^ mice died perinatally with severe craniofacial defect (complete cleft palate and shortened mandible) and spinal deformity. These findings suggest that LOXL3 is crucial for palatal and vertebral development in mice.

## Results

### Generation of mice with a targeted deletion of the ***Loxl3*** gene

**Figure 1.**
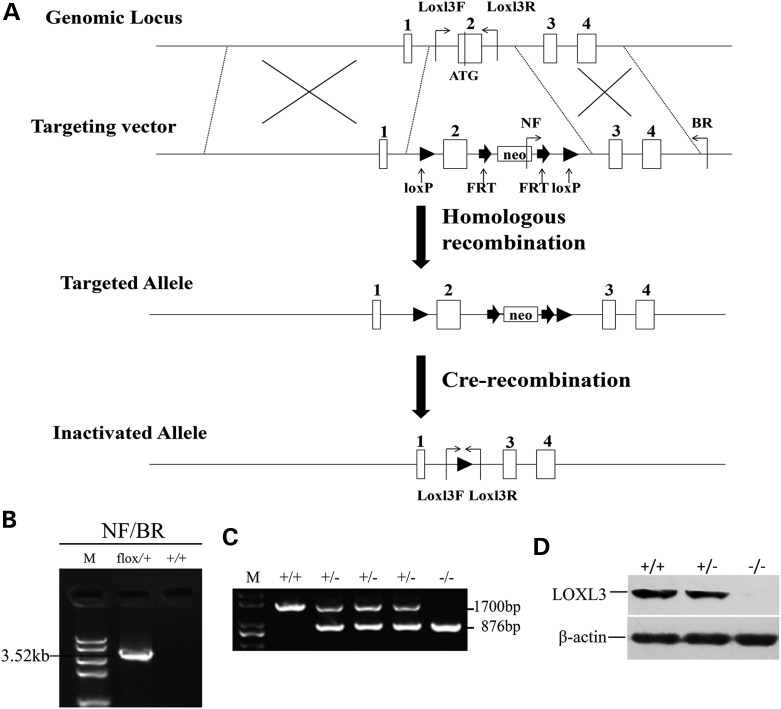
Targeted inactivation of the *Loxl3* gene. (**A**) Targeting strategy. Exons are numbered and depicted as white boxes. In the targeting construct, a loxP site was inserted upstream of exon 2 (containing the ATG start codon), and the FRT-neo-FRT-loxP cassette was inserted downstream of exon 2. (**B**) Identification of the targeted ES clones by long PCR with the indicated primers. Flox/+, targeted ES clone carrying the floxed *Loxl3* allele; +/+, wild-type ES clone. (**C**) Identification of mouse genotypes by PCR with the indicated primers. +/+, wild-type mice; +/−, *Loxl3^+/−^* mice; −/−, *Loxl3^−/−^* mice. (**D**) Western blot analysis for LOXL3 and β-actin of *Loxl3^+/+^*, *Loxl3^+/−^*and *Loxl3^−/−^* fetuses at E14.5.

The mouse *Loxl3* gene was inactivated by a two-step method consisting of homologous recombination in ES cells followed by Cre-recombination in mice (Fig. 1A). Homologous recombinants were identiﬁed by long PCR analyses (Fig. 1B). Deletion of exon 2 and the FRT-neo-FRT cassette by Cre-recombination led to the inactivation of the *Loxl3* gene. *Loxl3*^−/−^ mice were then generated by intercrossing *Loxl3*^+/−^ mice. (Fig. 1C). Western blotting confirmed the loss of LOXL3 protein expression in the whole fetus from embryonic day 14.5 (E14.5) (Fig. 1D).

### Targeted inactivation of the *Loxl3* gene resulted in a craniofacial defect and a spinal deformity

**Figure 2.**
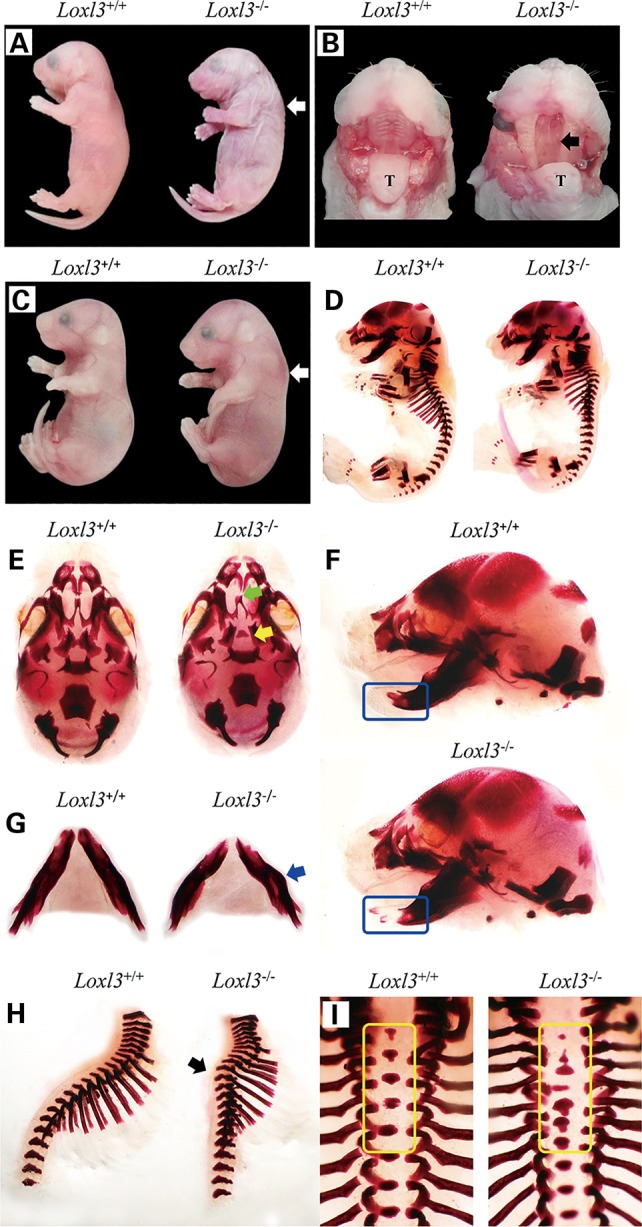
Abnormalities of *Loxl3^−/−^*mice. (**A** and **B**) deformity of the spine (white arrow) and cleft palate (black arrow) in newborn *Loxl3^−/−^* mice (P0). T: tongue. (**C–I**) Craniofacial and spinal defects of LOXL3-null mice at E18.5: (C) Spinal deformity (white arrow) of *Loxl3^−/−^* mice. (D) Lateral view of Alizarin Red staining of *Loxl3^−/−^* and control skeletons. (E) Ventral view of the skulls. The palatal shelves (yellow arrow) of *Loxl3^−/−^* mice were unfused and kept open. The vomer bones (green arrow) of *Loxl3^−/−^* mice were also distorted. (F and G) Lateral view (F) of the skulls and ventral view (G) of the mandible. The mandible of *Loxl3^−/−^* mice appeared shorter (blue rounded rectangle) compared with that of wild-type mice, and was bent (blue arrow). (H and I) Lateral view (H) and dorsal view (I) of the spine. LOXL3-deficient mice showed abnormal spine curvature (black arrow) and vertebral deformities (yellow rounded rectangle).

*Loxl3^+/−^* mice are normal and viable. There were no adult homozygous offspring found from *Loxl3^+/−^* crossings, raising the possibility that the ablation of *Loxl3* causes early lethality. *Loxl3^−/−^* newborns died shortly after birth, only14.2% survived at P0, and all of the homozygous mice died by P1 (Table 1). There was no obvious difference in the skin between homozygous mice and wild-type mice (Supplementary Material, Fig. S1). Newborn *Loxl3^−/−^* mice (P0) showed severe cleft palate and spinal deformity (Fig. 2A and B). All of the *Loxl3^−/−^* mice exhibited a cleft palate. Most of the *Loxl3^−/−^* mice (92.3%, 48/52) displayed a spinal deformity. Genotype analysis of offspring indicated that the numbers of wild-type, heterozygous and homozygous animals were distributed in a normal Mendelian ratio at embryonic stage E18.5 (Table 1). Craniofacial and spinal defects were already observed at E18.5 in *Loxl3^−/−^* mice (Fig. 2C–I). Skeletal staining clearly demonstrated craniofacial and spinal defects (Fig. 2D–I). At E18.5, the palatal shelves had already fused in wild-type mice, but the palatal shelves did not fuse and the vomer bone was distorted in *Loxl3^−/−^* mice (Fig. 2E). Abnormal mandibles were also observed in *Loxl3^−/−^* mice. The mandibles of *Loxl3^−/−^* mice were shorter and exhibited a bent shape compared with those of wild-type mice (Fig. 2F and G). LOXL3-deficient mice also displayed spine curvature disorders and deformities of vertebral bodies (Fig. 2H and I).

**Table 1.** Genotype frequency of the offspring from *Loxl3*^+/−^ mouse breedings

Stage	Number of animals (%) of genotype			Total number
	+/+	+/−	−/−	
E18.5	38 (25.2)	79 (52.3)	34 (22.5)	151
P0	39 (30.7)	70 (55.1)	18 (14.2)	127
P1	42 (36.8)	72 (63.2)	0 (< 1)	114

**Figure 3.**
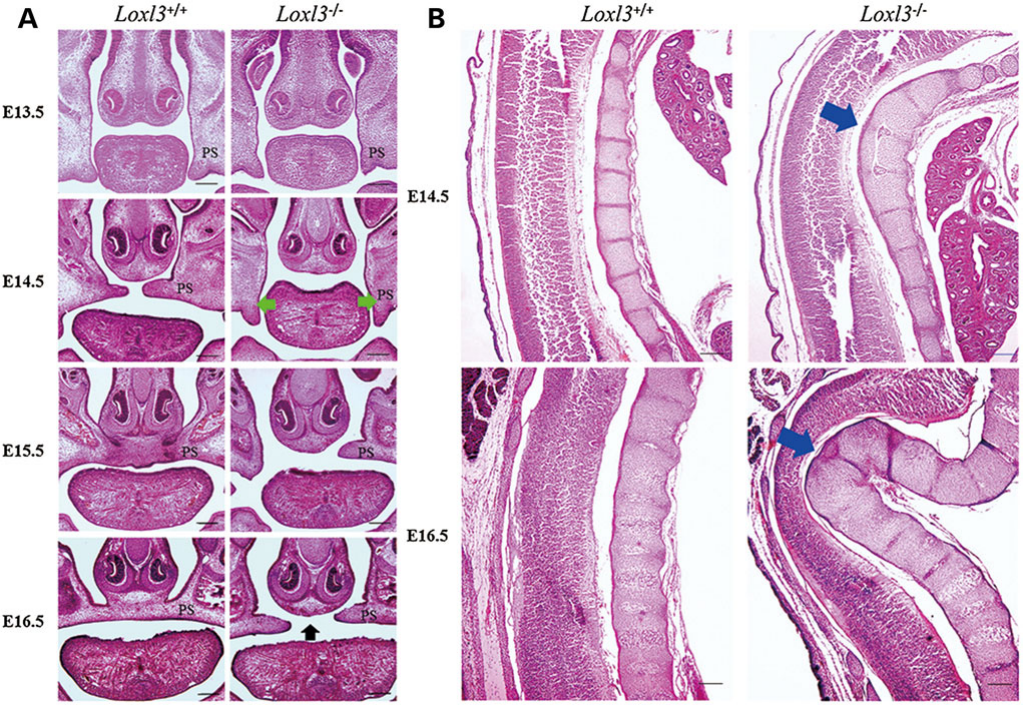
The changes in the palate shelves and thoracic vertebrae of *Loxl3^−/−^*mice at different embryonic time points. (**A**) Palatal shelves of *Loxl3^−/−^*mice at E14.5 failed to elevate (green arrows). The delayed palatal bone development continued until E16.5, and the palatal shelves did not contact and fuse (black arrow). (**B**) The cartilage primordia (future vertebral body) of the thoracic vertebrae became loose and underwent bending in *Loxl3^−/−^*mice (blue arrows) at E14.5 and E16.5. PS: palatal shelves. Bar: 200 μm.

Paraffin sectioning and H&E staining were performed to investigate the detailed development of the palate and the thoracic vertebrae. During the development of the palate in wild-type mice, the palatal shelves grew vertically at E13.5, and there was no significant difference between the *Loxl3*^−/−^ mice and the wild-type mice at this stage (Fig. 3A). At E14.5, the palatal shelf was elevated in wild-type mice but failed to elevate in *Loxl3*^−/−^ mice (Fig. 3A). We examined the cell proliferation and apoptosis of the palate at E14.5, and we found no obvious difference between the *Loxl3*^−/−^ mice and the wild-type mice (Supplementary Material, Figs. S2 and S3). Between the E15.5 and E16.5 stages, the palatal shelves adhered and fused in wild-type mice (Fig. 3A); However, the palatal shelves remained open and never completed the subsequent adhesion and fusion in *Loxl3*^−/−^ mice (Fig. 3A). The cartilage primordia (future vertebral body) develops from sclerotomes in wild-type mice. There is no obvious difference between *Loxl3*^−/−^ mice and wild-type mice until E13.5 (data not shown). At E14.5, the cartilage primordia of the thoracic vertebrae of *Loxl3^−/−^* mice enlarged and started bending (Fig. 3B). At E16.5, the bending of the thoracic vertebrae was very obvious and the spinal cord was squeezed in the *Loxl3^−/−^* mice (Fig. 3B).

**Figure 4.**
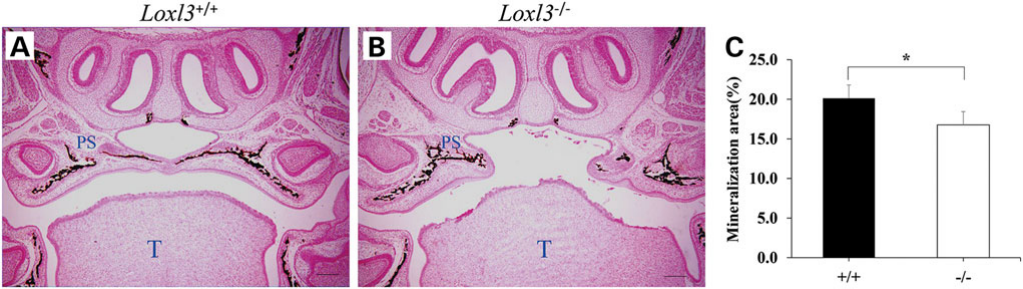
Mineralization in coronal sections of LOXL3-deficient palatal region by Von Kossa staining at E18.5. Mineralization appeared brown on the sections. (**A**) Wild-type mice. (**B**) LOXL3-deficient mice. (**C**)The mineralization area of palatal region of *Loxl3*^−/−^ mice was significantly less than that of *Loxl3^+/+^* mice. *P < 0.05. T: tongue; PS: palatal shelves. Bar = 200 μm.

To determine the pattern of mineralization during the formation of the secondary palate, tissue sections from the heads of E18.5 wild-type and mutant embryos were analysed with Von Kossa staining. Wild-type mice demonstrated normal intramembranous and endochondral ossification with mineral deposition in the secondary palate (Fig. 4A). Because the palatal shelves of mutant mice were unfused, the mineralization in the truncated palatal shelves was obviously decreased (*P* < 0.05, Fig. 4B and C).

### Histological analysis of eyes, heart, lung, aorta, trachea and diaphragm in LOXL3-deficient mice

**Figure 5.**
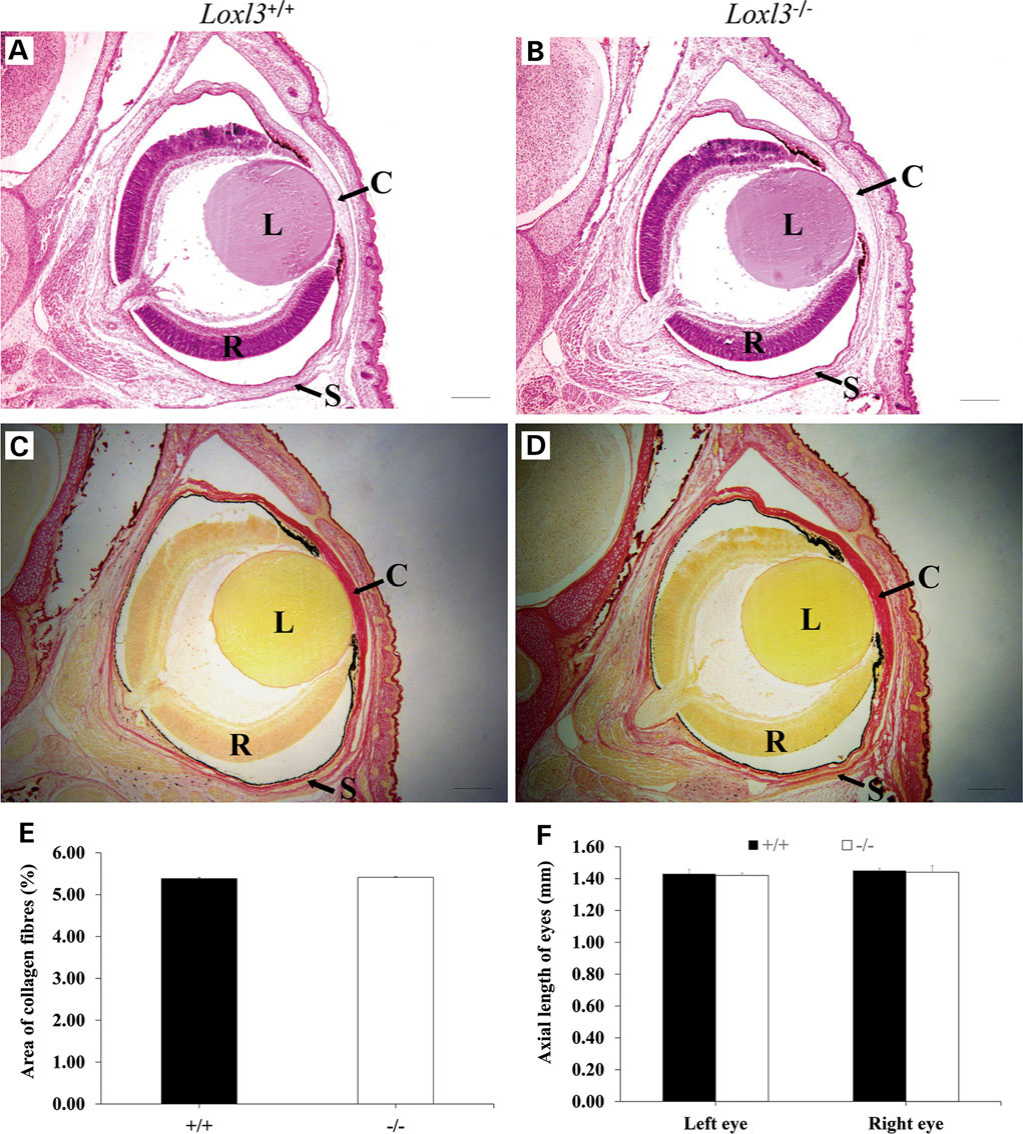
Histological analysis of eyes in LOXL3-deficient mice at E18.5. (**A** and **B**) H&E staining showed no apparent structural abnormality in eyes of LOXL3-deficient mice. (**C–E**) There was no obvious change in collagen despostion in cornea and sclera of LOXL3-deficient mice compared with that of wild-type mice. (**F**) No significant difference was found in the axial lengths of eyes between LOXL3-deficient mice and wild-type mice. C: Cornea; L: Lens; R: Retina;S: Sclera. Bar: 200 μm.

**Figure 6.**
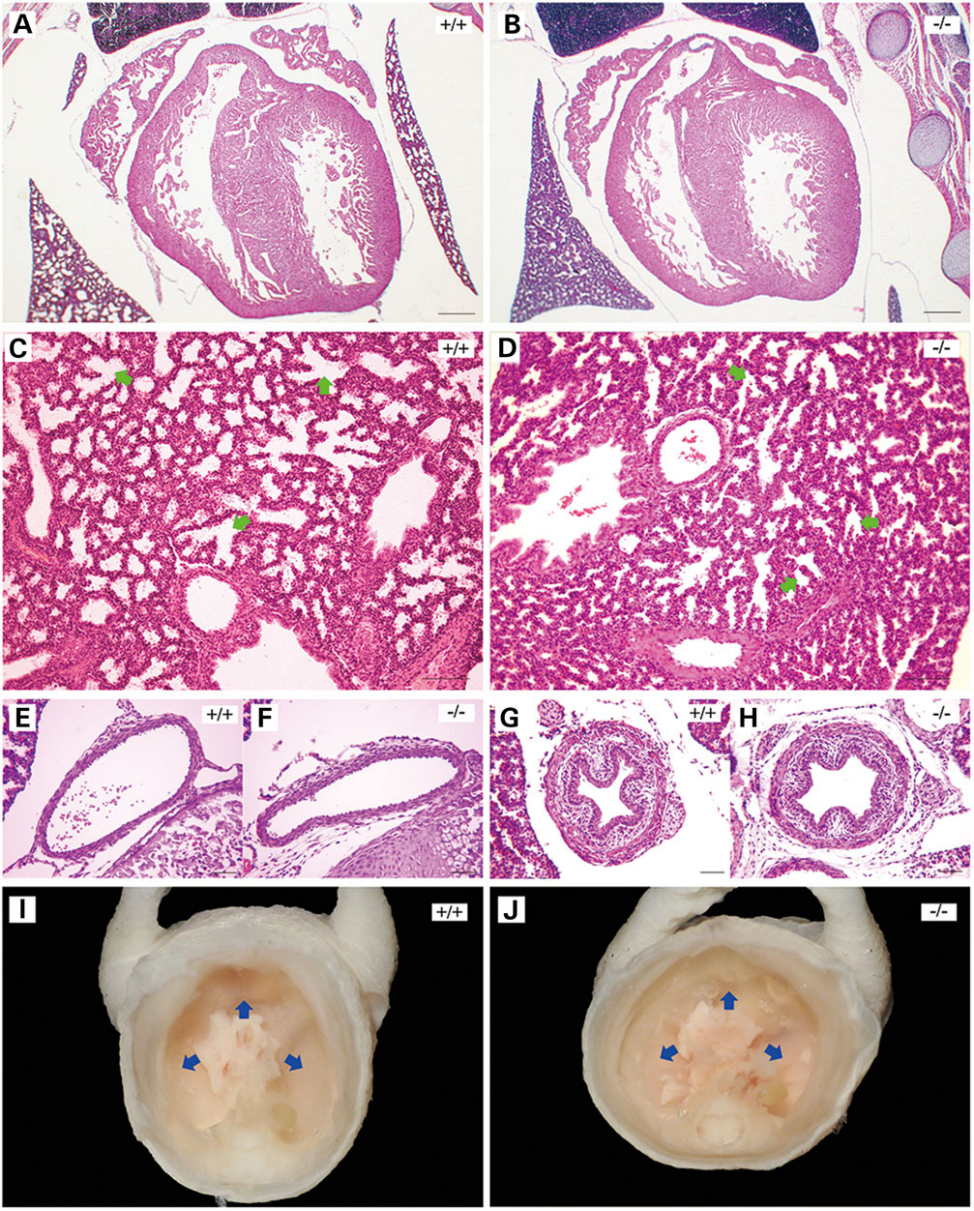
Morphological analysis of the heart, lung and aorta in LOXL3-deficient mice at E18.5. (**A** and **B**) There were no evident defects in the structure of heart in LOXL3-deficient mice. Bar: 200 μm. (**C** and **D**) The alveoli (green arrows) of LOXL3-deficient lungs were smaller than those of wild-type mice. Bar: 100 μm. The aortas (**E** and **F**) and tracheae (**G** and **H**) were normal in LOXL3-deficient mice. Bar: 50 μm. (**I** and **J**) Ventral view of thorax in mice. LOXL3-deficient mice had intact diaphragms (blue arrows).

Patients with Stickler syndrome caused by human *LOXL3* mutation present with symptoms of high non-progressive myopia (14). Based on this phenotype in humans we examined the morphology of the eyes in mutant mice at E18.5. The structure of eyes in LOXL3 knockout mice was normal (Fig. 5A and B). Sirius red staining showed that collagen was mainly deposited on the cornea and sclera of eyes (Fig. 5C and D). The distribution of collagen fibres in cornea and sclera displayed no apparent difference between LOXL3 knockout and wild-type mice (Fig. 5E). We also examined the axial length of eyes in mice and there was no significant difference between the two groups (Fig. 5F). Hearts, aortas and tracheae of LOXL3 knockout mice exhibited no obvious abnormalities (Fig. 6A, B, E, F, G and H). Blood vessels and bronchi in lung of LOXL3-deficient mice were normal, while the alveoli of LOXL3-deficient lungs was smaller than those of the wild-type mice (Fig. 6C and D). Although thorax deformity as a result of abnormal curvature of the spine was observed, LOXL3-deficient mice still possessed intact diaphragms (Fig. 6I and J).

### The expression of *Loxl3* in the palate, spine and eyes of mice

**Figure 7.**
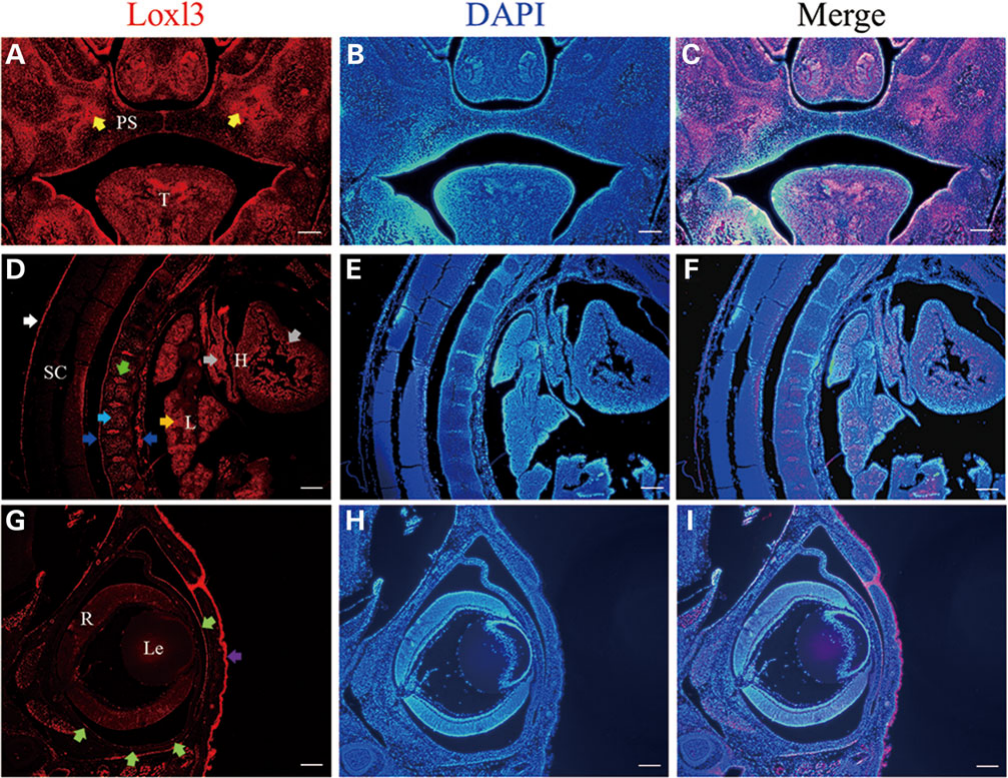
Localization of LOXL3 protein in the palate, spine and eyes of mice. The expression of *Loxl3* was checked by immunofluorescence staining. DAPI was used for nuclear staining. (**A–C**) The expression of *Loxl3* was mainly localized to the palate mesenchyme (yellow arrows) and tongue in wild-type mice at E14.5. (**D–F**) LOXL3 protein was expressed in the original intervertebral disc (green arrow), cartilage primordia (light blue arrow), anterior and posterior longitudinal ligaments (blue arrows), meninges of spinal cord (white arrow), lung (orange arrow) and heart (grey arrows) at E14.5. (**G–I**) LOXL3 protein was also weakly expressed in cornea and sclera (light green arrows) and strongly expressed in the skin of the eyelid (purple arrow) of eyes at E18.5. T: tongue; PS: palatal shelves; SC: spinal cord; L: lung; H: heart; Le: Lens; R: Retina. Bar: 200 μm.

Because cleft palates and spinal deformities were observed in LOXL3-deficient mice, we firstly examined the expression of *Loxl3* in palate and spine. Immunofluorescence staining was performed on coronal sections from the anterior region of the palate (Fig. 7A–C) and sagittal sections of the spinal columns (Fig. 7D–F) at E14.5. The results showed that LOXL3 protein was predominantly expressed in the palate mesenchyme and tongue (Fig. 7A). We also examined the expression of *Loxl3* in the palate at different embryonic stages, and the expression of *Loxl3* was detected as early as E12.5, a stage before the palates fused (Supplementary Material, Fig. S4). LOXL3 protein was expressed in the original intervertebral disc, cartilage primordia, anterior and posterior longitudinal ligaments, meninges of spinal cord, lung and heart (Fig. 7D). In addition to palate and spine, we also examined the expression of *Loxl3* in eyes at E18.5 (Fig. 7G–I). LOXL3 protein was strongly expressed in the skin of the eyelid and weakly expressed in the cornea and sclera (Fig. 7G).

### Collagen fibre abnormality in palate shelves and cartilage primordia of the thoracic vertebrae

**Figure 8.**
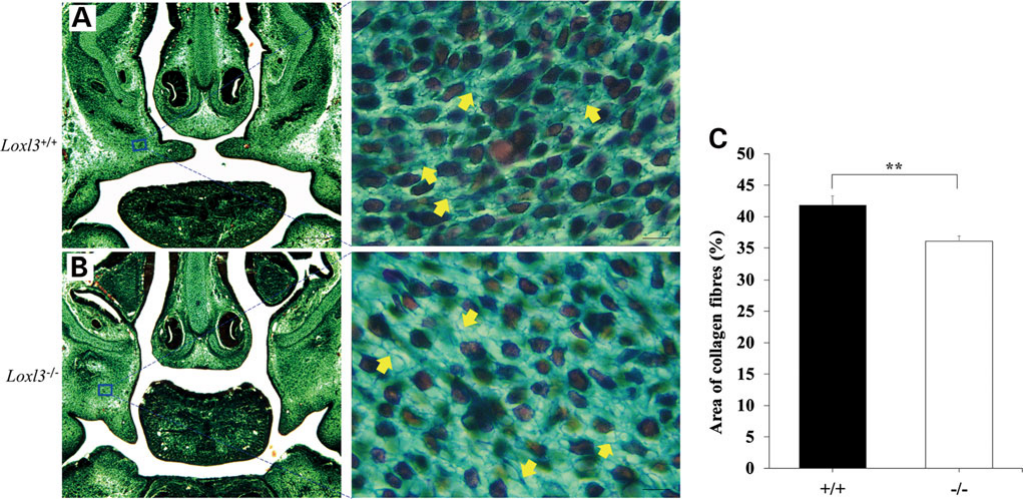
A decrease in collagen density of palate shelves in *Loxl3^−/−^* fetuses at E14.5. (**A**) Wild-type mice. (**B**) LOXL3-deficient mice. Collagen fibres (yellow arrows) in sections with Masson's trichrome staining under a light microscope. (**C**) The collagen fibre density of palate shelves in *Loxl3^−/−^* mice revealed a significant decrease compared with that in *Loxl3^+/+^* mice. ** *P* < 0.01. Left bar: 200 μm. Right bar: 10 μm.

**Figure 9.**
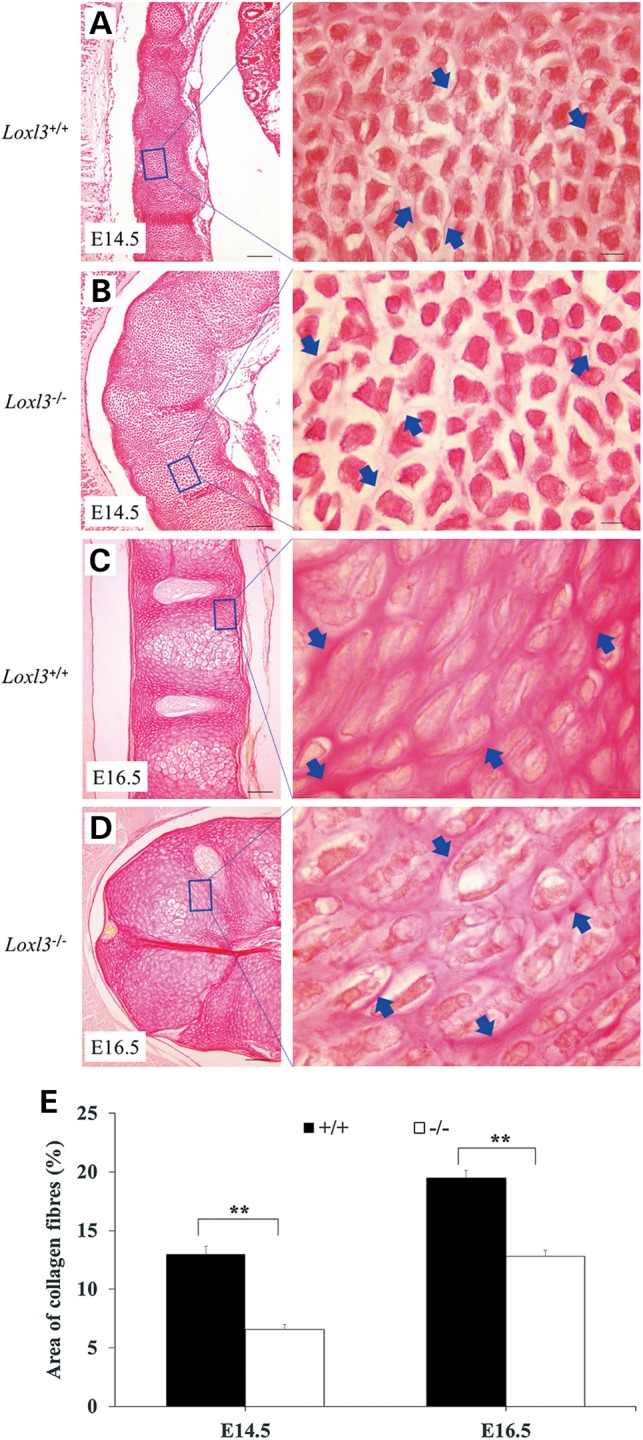
Collagen defect in the cartilage primordia of the thoracic vertebrae of *Loxl3^−/−^* fetuses (Sirius red staining). (**A** and **B**) In LOXL3 knockout mice at E14.5, chondrocytes in the cartilage primordia were loosely organized because of a reduction in the collagen fibres around the chondrocytes. (**C** and **D**) Collagen fibres around the columnar chondrocytes in LOXL3 knockout mice were still sparse at E16.5. (**E**) Whether at E14.5 or at E16.5, the collagen fibre density around the chondrocytes in *Loxl3^−/−^* mice was significantly less than that in *Loxl3^+/+^* mice. **P < 0.01. Blue arrows: collagen fibres. Left bar: 100 μm. Right bar: 10 μm.

Mature collagen fibres consist of multiple collagen fibrils. Collagen fibrils are formed from multiple tropocollagen molecules through covalent cross-linking catalysed by LOXs. Thus we examined the collagen fibres in palate shelves and cartilage primordia of the thoracic vertebrae. Masson's trichrome staining showed that collagen fibres in *Loxl3^−/−^* palate shelves compared with *Loxl3^+/+^* palate shelves were significantly decreased at E14.5 (*P* < 0.01, Fig. 8). The thoracic vertebrae of *Loxl3^−/−^*mice started bending at E14.5, and this phenotype became very obvious around E16.5. Sagittal sections and Sirius red staining were used to check the collagen fibres in the thoracic vertebrae at E14.5 and E16.5. The chondrocytes in the cartilage primordia of the thoracic vertebrae were more dispersed in *Loxl3^−/−^*mice than in wild-type mice at E14.5 (Fig. 9A and B). Sparse collagen fibres around the columnar chondrocytes were also observed in *Loxl3^−/−^* mice at E16.5 (Fig. 9C and D). The amount of collagen fibres around the chondrocytes was significantly decreased in *Loxl3^−/−^*mice at E14.5 or E16.5 (*P* < 0.01, Fig. 9E).

### Alterations in collagen cross-links

**Figure 10.**
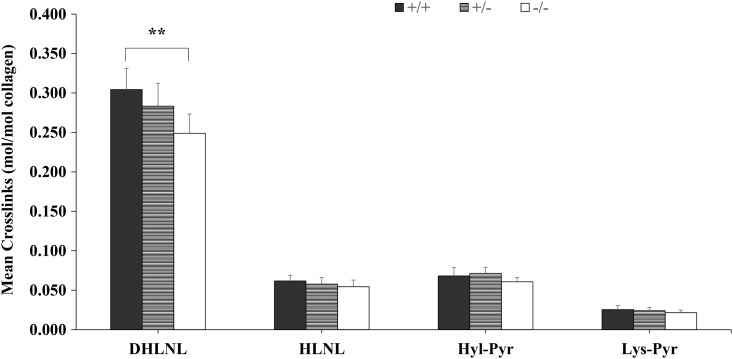
The quantification of collagen cross-links. The concentration of DHLNL was significantly less than wild-type mice (+/+) for homozygous knock out mice (−/−), but there was no significant difference in HLNL, Hyl-Pyr and Lys-Pyr levels among the different groups. ** represents *P* < 0.01.

To investigate the effect of LOXL3 on collagen cross-links, we examined the collagen cross-links in eviscerated embryos with craniofacial bones and spines at E14.5. The immature collagen cross-links, dehydrohydroxylysinonorleucine (deH-HLNL) and hydroxylysino-keto-norleucine (HLKNL), were unstable and reduced to hydroxylysinonorleucine (HLNL) and dihydroxylysinonorleucine (DHLNL), respectively (17). The immature collagen cross-links that reduced can be detected. The mature collagen cross-links, hydroxylysylpyridinoline (HYL-PYR) and lysylpyridinoline (LYS-PYR), are stable and detected directly. The concentration of DHLNL was significantly decreased in *Loxl3^−/−^* mice (*P* < 0.01, Fig. 10). The concentrations of HLNL, HYL-PYR and LYS-PYR in *Loxl3^−/−^* mice were also less than those of *Loxl3^+/+^* mice, but these differences were not obvious (Fig. 10). There was also no significant difference between *Loxl3^+/−^* and *Loxl3^+/+^* mice in the concentrations of DHLNL, HLNL, HYL-PYR or LYS-PYR (Fig. 10). However, the concentration of DHLNL was decreased in *Loxl3^+/−^* mice compared with *Loxl3^+/+^* mice.

### Hydroxyproline and desmosine changes

**Figure 11.**
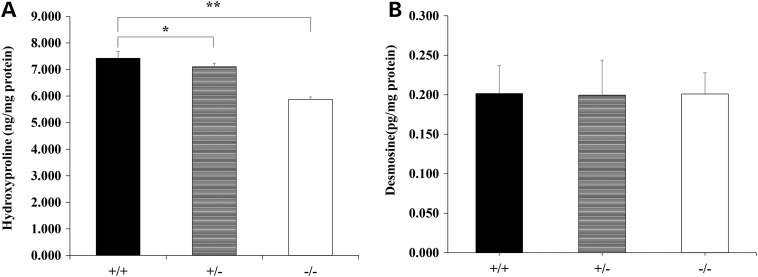
Hydroxyproline and desmosine changes. Eviscerated embryos with craniofacial bones and spines were detected at E14.5. (**A**) The hydroxyproline assay demonstrated that the hydroxyproline content of *Loxl3^−/−^* mice compared with *Loxl3^+/+^ mice* was significantly decreased (*n* = 5 in each group, mean ± SD, **P < 0.01). For *Loxl3^+/−^* mice, the hydroxyproline content was also less than that of *Loxl3^+/+^ mice* (*n* = 5 in each group, mean ± SD,*P < 0.05). (**B**) The desmosine concentration was analysed by ELISA. No significant difference was found among the three groups (*n* = 5 in each group).

To examine the amount of collagen fibres, a hydroxyproline assay was used to quantify the collagen content in eviscerated embryos at E14.5. The hydroxyproline content of *Loxl3^−/−^* mice was significantly less than that of *Loxl3^+/+^* mice (*P* < 0.01, Fig. 11A). A decrease of the hydroxyproline content in *Loxl3^+/−^* mice was also observed (*P* < 0.05, Fig. 11A). We also examined the concentration of desmosine, a cross-linking amino acid of mature elastin. There was no significant difference in desmosine concentrations among the three groups (Fig. 11B).

## Discussion

Many studies have been performed to reveal the functions of LOX and LOXL1, but the probable roles of other LOX family members remain unknown (10–12). LOXL3, one of the LOX family members, is different from LOX or LOXL1 and is more similar to LOXL2 and LOXL4 in structure. LOXL3-deficient mice were generated by a gene targeting strategy, and the mutant mice died rapidly after birth. Moreover, the loss of LOXL3 resulted in severe craniofacial (cleft palate and shortened mandible) and spinal defects. In the current study, all of the homozygous knockout mice showed a cleft palate and shortened mandible, and approximately 92.3% of the homozygous knockout mice displayed a spinal deformity. To our knowledge, this is the first demonstration that LOXL3 is associated with palate and spinal development. It has been previously reported that cleft palate is often associated with perinatal death (18,19). Therefore, we theorized that there exists a causal link between cleft palate and perinatal death in our LOXL3 knockout mice.

Palatogenesis is an important developmental process, and it can be disrupted by genetic perturbations (20). Cleft palate is a serious consequence of disrupted palatogenesis. Cleft palate is the fourth most common human birth defect in the United States, affecting approximately 1 out of every 700 live births (NIDCD, 2008). The cause of cleft palate is mostly unknown, and the condition appears to be multifactorial. Palate development is a multistep process that involves palatal shelf growth, elevation, adhesion and fusion (20). Disruption in any step is likely to induce a cleft palate. In our LOXL3 knockout mice, the cleft palate abnormality was first observed around E14.5. The palatal shelf did not follow the normal developmental procedure and failed to elevate at E14.5. The precise mechanisms of palatal shelf elevation remain unclear, but collagen may play a significant role in shelf elevation (21). Procollagen is first synthesized in the cells and is then secreted into the extracellular matrix. Procollagen is transformed into tropocollagen by procollagen peptidase. Multiple tropocollagen molecules form collagen fibrils through covalent cross-linking by LOXs. Collagen cross-linking can improve the stability of mature collagen fibrils (22). A previous study reported that the inhibition of collagen cross-links in the rat embryo by β-aminopropionitrile (BAPN), a naturally occurring inhibitor of LOXs, could induce cleft palate (23). In the current study, LOXL3 was found to be highly expressed in the palatal mesenchyme. We speculated that LOXL3 may play important roles in the assembly of collagen by catalyzing collagen cross-linking in the palatal mesenchyme. In the LOXL3-deficient mice, different levels of reduction occurred in immature cross-links (DHLNL and HLNL) and mature cross-links (HYL-PYR and LYS-PYR). Particularly, DHLNL was significantly decreased in the LOXL3-deficient mice compared with the wild-type mice. We knew that the immature cross-link in cartilage and bone was predominantly DHLNL (17). Moreover, it has been suggested that LOXL3 has a high amine oxidase activity in different collagen types and has a possible functional role in bone development or maintenance in humans (13). In the normal development of the palate, palatal shelf elevation is associated with increased secretion of collagen, which is believed to strengthen the elasticity of the palatal shelf and maintain the integrity of the palate (24). If a decrease in collagen secretion occurs, it is very likely that palatal shelf elevation cannot be completed. In our study, we also found that the density of collagen fibre and the amount of collagen were reduced in LOXL3-deficient palates. Taken together, the lack of LOXL3 resulted in the decrease of collagen cross-links and collagen content, which lead to the failure of palatal shelf elevation. The failure of palatal shelf elevation resulted in the formation of cleft palate.

In addition to cleft palate, most of the LOXL3 knockout mice also showed a spinal deformity, specifically an abnormal backward bending of the vertebral column. The spine bent at E14.5 and became more severe over time. In our study, we demonstrated that LOXL3 affected collagen assembly directly. The vertebral column of mice is rich in collagen and different types of collagen had been identified, including collagens type I, II, III, IX, X and XI (25). In previous studies, different types of collagen mutations have been demonstrated to result in abnormal curvature of the spine. Mice with a dominant mutation in a splice donor of the collagen alpha1 type I gene (*col1a1^Jrt^/+*) displayed traits associated with Ehlers–Danlos syndrome (EDS), and a third of the young adult mice had noticeable curvature of the spine (26). Deletion of Type V collagen in the mouse also resulted in spinal deformity, which was caused by disorganized type I collagen fibrils (27). Moreover, loss of collagen type VIII alpha1a (*Col8a1a*) function resulted in congenital vertebral malformations during zebrafish embryogenesis (28). In our study, spinal deformity caused by the lack of LOXL3 was similar to those caused by collagen mutations, but by no means identical. In LOXL3-deficient mice, an obvious decrease in collagen cross-links was detected. In a previous study, BAPN (LOXs inhibitor) treatment inhibited collagen cross-links in the spinal column and the surrounding longitudinal ligaments of rat fetuses, and then resulted in a severe spinal deformity and spinal cord lesion (29). Thus, we have reason to believe that the reduction in collagen cross-links that are induced by the lack of LOXL3 resulted in spinal deformities in mice. We also found that a decrease in the collagen content was detected and sparse collagen fibres around the chondrocytes of the thoracic vertebrae were observed. We speculated that the reduced collagen fibres failed to support the normal development of the spine in LOXL3-deficient mice. However, in patients with a missense variant in human *LOXL3* gene, spinal deformity was undetected (14). We suggested that the human *LOXL3* mutation was only a missense mutation, but we produced a complete LOXL3 knockout mice. As a result, the difference in spinal phenotype is plausible.

In a previous study, in addition to cleft palate, myopia is also very severe in patients with Stickler syndrome (14). In our study, we did not observe apparent defects in the eyes of our mutant mice at E18.5. We examined the expression of *Loxl3* gene in the eyes of mice, and we found that its levels were relatively low at E18.5. This may explain the normal status of mutant mouse eyes at embryonic period. We analysed our mutant mice at E18.5, which was still in the early stages of eye development. The eyes of mice continued to develop after birth until full maturity. Since our mutant mice died perinatally, it is uncertain whether LOXL3-deficient mice still maintain normal eye development after birth. It is important to make a conditional knockout mice by using an eye specific Cre and our floxed *Loxl3* mice and study the functions of LOXL3 in the development of eyes after birth. Hearts, aortas, tracheae and diaphragms of mutant mice showed no apparent abnormalities, which were different from the phenotype in the mice of LOX deletion (10), indicating the differing functions of LOX and LOXL3. We also found that the alveoli of lung in mutant mice were smaller than those in wild-type mice. It is unclear whether or not the change will interfere with the normal function of the lungs, and more testing needs to be done.

Of the five LOXs, LOX and LOXL1 are homologous, while LOXL2, LOXL3 and LOXL4 are similar. In prior studies (10–12), the functions of LOX and LOXL1 have been shown to be different. LOX catalyses the cross-linking of collagen as well as elastin, while LOXL1 mainly serves as a cross-linking enzyme of elastin. In our study, a significant decrease in collagen-links from the lack of LOXL3 caused cleft palate and spinal deformity, while no obvious difference was observed in the elastin cross-links in the palate and spine. We noticed that the collagen cross-links and the amount of collagen in LOXL3 heterozygous knockout mice was also reduced, but these mice were normal and viable. One possible explanation is that the minimal decrease of collagen cross-links and collagen content in LOXL3 heterozygous knockout mice is insufficient to induce cleft palate and spinal deformity. The deletion of LOXL3 did not cause a total loss of the amount of collagen, indicating that other LOX family members must be involved in the assembly of collagen. Moreover, cleft palate and a spinal deformity were not reported in LOX or LOXL1 knockout mice. Thus, LOXL3, instead of LOX or LOXL1, plays an irreplaceably important role in the normal development of the palate and spine. Different phenotypes in the LOX and LOXL1 knockout mice have been reported. In our study, LOXL3 was found to have unique functions in the palate and spine. Thus, despite similar structures, LOX family members have different functions *in vivo*. Future studies of LOXL2 and LOXL4, possibly by gene targeting, will reveal additional functions of the LOX gene family.

*LOXL3* has already been identified as a candidate human disease gene*.* Patients with the missense variant in human *LOXL3* gene, which is believed to cause autosomal recessive Stickler syndrome, exhibit conditions of micro/retrognathia, cleft palate, myopia and mild conductive hearing loss (14). In humans, Stickler syndrome caused by mutations of *COL2A1*, *COL11A1*, *COL11A2*, *COL9A1* and *COL9A2*, is one of the most common connective tissue disorders characterized by ocular, skeletal, orofacial and auditory defects (15,16,30). Type II and XI collagens represent the major collagens in Stickler syndrome pathogenesis. In previous studies, mouse models with different mutations of *COL2A1* gene have been investigated (15,31–34). These mutant mice displayed severe chondrodysplasia, craniofacial deformities, cleft palate and neonatal death with no reported abnormalities of the eyes. These phenotypes were similar to those of our LOX3-deficient mice. Targeted inactivation of the *COL2A1* gene resulted in the closure of the alveoli of the lungs and mutant mice died of acute respiratory distress (31,34). Our LOX3-deficient mice also experienced mild alveolar shrinkage. Whether or not this change causes respiratory distress remains unclear. Like *COL2A1* gene, *COL11A1* gene mutant mice had phenotypic changes of neonatal lethality, cleft palate, small mandible, small thorax, disproportionate limbs and fragile cartilage (35). LOXL3 knockout mice, which was different from *COL2A1* and *COL11A1* gene mutant mice, presented with spinal deformity but no dwarfism characteristic of chondrodysplasia. These differences suggested that LOXL3 catalyses not only type II and XI collagens but also other types of collagens. Moreover the loss of Loxl3b as an ortholog of mammalian LOXL3 in zebrafish, has been shown to lead to craniofacial defects (36). This study corroborates with our findings that is suggestive of an important role of LOXL3 in proper craniofacial morphogenesis. Except for Stickler syndrome, as a candidate human disease gene, mutations in LOXL3 may also be screened in other patients with cleft palate or spinal deformity. In future studies, we may conditionally knockout the *Loxl3* gene in specific tissues or knock-in the *LOXL3* human mutation to obtain viable mice in which cleft palate or spinal deformity is still present. This type of mouse may be used as an animal model of human disease.

## Materials and Methods

### *Loxl3*^−/−^ gene targeting

The BAC clone containing the *Loxl3* gene (bMQ412o17, Source Bioscience) was identified in the mouse BAC (bMQ) Library. An 8.84-kb genomic fragment containing exons 1–4 of the *Loxl3* gene was retrieved from the BAC clone to produce the targeting vector as previously described (37,38). A loxP site was inserted upstream of exon 2, and the FRT-neo-FRT-loxP cassette was inserted downstream of exon 2 in the targeting vector (Fig. 1A). Exon 2 containing the ATG start codon can be deleted by the Cre-*loxP* system. The deletion of the ATG start codon will prevent the translation of *Loxl3* mRNA and cause the loss of the LOXL3 protein. The linearized targeting vector was electroporated into 129/SvEv (129S6) ES cells, and neo-resistant ES cell clones were selected by G418. The positive ES cell clones that underwent homologous recombination were screened by PCR. A forward primer of neo (NF: 5′-TCGCCTTCTTGACGAGTTCT-3′) and a reverse primer outside of the short arm (BR: 5′-GCGGTGATTATCTGGATTTTAGC-3′) were used to screen for the targeted ES clones. The clones with a 3.52-kb band were the targeted ES cells (Fig. 1B). The targeted ES cells were microinjected into C57BL/6J blastocysts. The chimeras were crossed with C57BL/6J mice, and germline transmitted mice were obtained. Mutant mice (*Loxl3*^flox*/+*^) carrying the floxed *Loxl3* allele were crossed with EIIa-Cre mice (ubiquitous Cre activity). Because of the mosaic activities of Cre recombinase, the first offspring of the EIIa-Cre; *Loxl3*^flox*/+*^ mice might be chimeric with different deletions. Therefore, the chimeric offspring were backcrossed with C57BL/6J to generate *Loxl3*^+/−^ mice, which were then intercrossed for the production of LOXL3-deficient mice (*Loxl3*^−/−^). The primers used for mouse genotyping are as follows: loxl3-F (5′-TGCTGTCATTTCTCCTGTGC-3′) and Loxl3-R (5′-ATTGAACCCAACCGTCATGT-3′) for the null and the wild-type alleles. An 876-bp or a 1700-bp fragment was produced from the null allele or the wild-type allele, respectively. All animal protocols were approved by the Animal Ethics Review Committee of Shandong University.

### Histological analysis

Embryos were isolated at different stages, fixed overnight in 10% neutral buffered formalin and embedded in paraffin. Coronal sections of hearts and transverse sections of lungs, aortae and tracheae were stained with haematoxylin and eosin (H&E). Coronal sections of the anterior region of palate and eyes and sagittal sections of spinal columns were stained with H&E, Sirius red or Masson's trichrome, Photomicrographs of Sirius red (Ruitaibio) or Masson's trichrome (Yuanyebio) were analysed with NIH image analysis software (ImageJ Version 1.48 V, National Institutes of Health, USA). The area of collagen fibres was calculated as the ratio of the integral optical density of collagen fibres to the total area.

For skeletal staining, skinned and eviscerated fetuses at E18.5 were fixed overnight in 10% neutral buffered formalin. Before staining, embryos were treated with 1% KOH for 2 days until they became clear. Bone staining was carried out by transferring embryos to a freshly made 0.1% alizarin red S (Sigma-Aldrich) for 2 days. Embryos were rinsed three times in 1% KOH (several hours each time). Finally, stained bones were treated through a gradient series of glycerol-KOH. Von Kossa staining of the palate at E18.5 was performed by Von Kossa calcium staining kit (GENMED SCIENTIFICS INC. USA). Analysis of images was carried out using Image Pro Plus 6.0 software. The mineralization area was calculated as the ratio of calcium staining area to the total observed area.

### Axial length measurement

The axial length of the eyes was measured by A-scan ultrasonography (ODM-1000A/P) with a 10-MHz transducer. Pups were anaesthetized and eyelids were stripped. The axial length of the eyes was defined as the distance from the front of the cornea to the back of the sclera.

### Antibodies, western blotting and immunofluorescence

Western blotting of craniofacial and spinal column tissue homogenates from fetuses was performed according to standard protocols. The primary antibodies that were used were rabbit anti-LOXL3 (1:400, 20–5193; American Research Products, Inc., Waltham, USA) and rabbit anti-β-actin antibodies (1:5000, P30002, Abmart). The secondary antibody that were used was goat anti-rabbit IgG-HRP (1:5000, ZB-2301; ZSGB-BIO). We preformed immunofluorescence staining on sagittal sections of the spinal columns and coronal sections from the anterior region of the palates from fetuses. The primary antibody that was used was rabbit anti-LOXL3 (1:400, 20–5193; American Research Products, Inc., Waltham, USA).The secondary antibody that was used was fluorescein-conjugated affinipure goat anti-rabbit IgG (H+L) (1:200; ZF-0311; ZSGB-BIO).

### Cell proliferation and apoptosis assay

Pregnant mice were injected with BrdU (sigma) at 100 μg/g of body weight. Embryos were fixed with 4% paraformaldehyde, embedded in OCT and sectioned at 10 μm. BrdU detection was performed according to the manufacturer's manual (Sigma). Apoptosis was examined via the TUNEL assay using an *in situ* cell death detection kit (Roche) with a modified protocol based on the manufacturer's instructions.

### Collagen cross-link assay

To determine the effect of LOXL3 on the synthesis and cross-linking of embryonic collagen, we examined the cross-linking of embryonic collagen as previously described (17). Mice were sacrificed at E14.5. Individual eviscerated embryo samples with craniofacial bones and spine, were homogenized and suspended in phosphate-buffered saline (0.15 M sodium chloride, 0.1 M sodium phosphate buffer, pH 7.4) and then reduced with sodium borohydride. Freeze-dried samples were hydrolysed in 6 N HCl at 110°C for 24 h. The hydrolysate was eluted from a cellulose column (Sigma) to separate the cross-linking amino acids from the standard amino acids. The cross-linking amino acids were then quantified using an amino acid analyser (L-8900, Hitachi High Technologies America, Inc., JP) using a modified gradient procedure.

### Hydroxyproline and desmosine assay

Mice were sacrificed at E14.5. Individual eviscerated embryos with craniofacial bones and spine were weighed and homogenized. Hydroxyproline is a major component of the protein collagen. The hydroxyproline content accurately reﬂects the amount of collagen in the sample. The sample was hydrolyzed and hydroxyproline was quantified by a hydroxyproline assay kit (Nanjing Jiancheng Bioengineering Institute, CN). Desmosine, a cross-linking amino acid of mature elastin, can be used to express elastin content. Desmosine was quantified by an ELISIA kit as previously described (39). Desmosine ELISA kit were purchased from Cusabio Biotech (Wuhan, CN) and performed according to manufacturer's instructions.

### Statistical analysis

Data are expressed as the mean ± SD. Differences in the measured variables between experimental and control groups were assessed using Student's *t*-test. Differences were considered statistically significant at *P* < 0.05.

## Supplementary Material

Supplementary Material is available at *HMG* online.

## Funding

This work was supported by National Natural Science Foundation of China Grant numbers 30871436, 30973297 and 31171194, National Basic Research Program (973) of China Grant numbers 2010CB945002 and 2014CB541703, Shandong Provincial Science and Technology Key Program Grant number 2009GG10002039, Shandong Provincial Natural Science Foundation of China Grant number ZR2013CQ041 and Independent Innovation Foundation of Shandong University Grant number 2013GN011. Funding to pay the Open Access publication charges for this article was provided by the National Basic Research Program (973) of China Grant number 2014CB541703.

## Supplementary Material

Supplementary Data
