# Supplementary material for: Loss of lysyl oxidase-like 3 causes cleft palate and spinal deformity in mice
Source: Hum Mol Genet. 2015 Aug 24;24(21):6174–85. doi: 10.1093/hmg/ddv333 (PMC4599675; doi:10.1093/hmg/ddv333)
Supplement: Supplementary Data [file supp_24_21_6174__index.html]

Loss of lysyl oxidase-like 3 causes cleft palate and spinal deformity in mice — Loss of lysyl oxidase-like 3 causes cleft palate and spinal deformity in mice — Supplementary Data 

# Loss of lysyl oxidase-like 3 causes cleft palate and spinal deformity in mice

## Supplementary Data

Supplementary Data

- Supplementary Data - Doc file
