# Supplementary material for: Loss of lysyl oxidase-like 3 causes cleft palate and spinal deformity in mice
Source: Hum Mol Genet. 2015 Aug 24;24(21):6174–85. doi: 10.1093/hmg/ddv333 (PMC4599675; doi:10.1093/hmg/ddv333)
Supplement: Supplementary Data [file supp_ddv333_ddv333supp.doc]

Loss of lysyl oxidase-like 3 causes cleft palate and spinal deformity in mice

Jian Zhang, Rui Yang, Ziyi Liu, Congzhe Hou, Wen Zong, Aizhen Zhang, Xiaoyang Sun, Jiangang Gao*

Institute of Developmental Biology, School of Life Science, Shandong University, 27 Shanda Nanlu, Jinan 250100, China.

* To whom correspondence should be addressed at: Institute of Developmental Biology, School of Life Science, Shandong University, 27 Shanda Nanlu, Jinan 250100, China. Tel: +86-531-88365399; Fax: +86-531-88363772; Email: jggao@sdu.edu.cn.


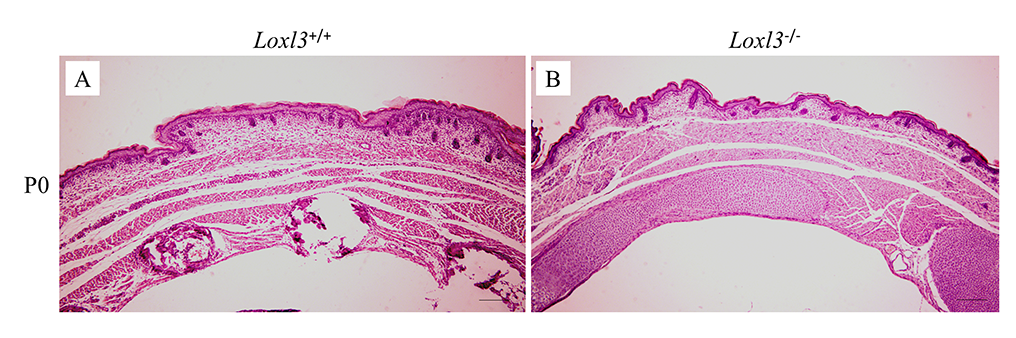


Supplementary Figure S1. H&E staining of skin from wild-type (A) and mutant mice (B) at P0. There was no obvious difference in skin between mutants and controls. Bar:200μm.


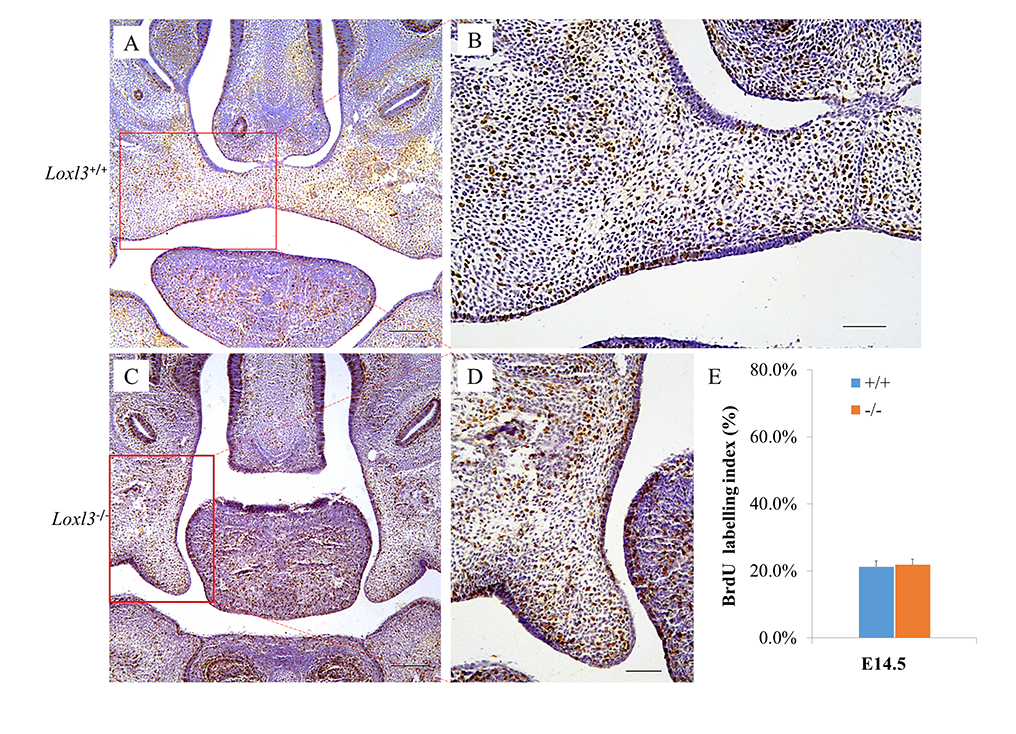


Supplementary Figure S2. BrdU immunohistochemistry in palate shelves of wild-type (A and B) and mutant (C and D) embryos at E14.5. (E) Quantification of BrdU positive cells within palate shelves at E14.5 (n=3 mice per genotype). There was no obvious difference between mutants and controls. Bar:A and C, 200μm; B and D, 80μm.


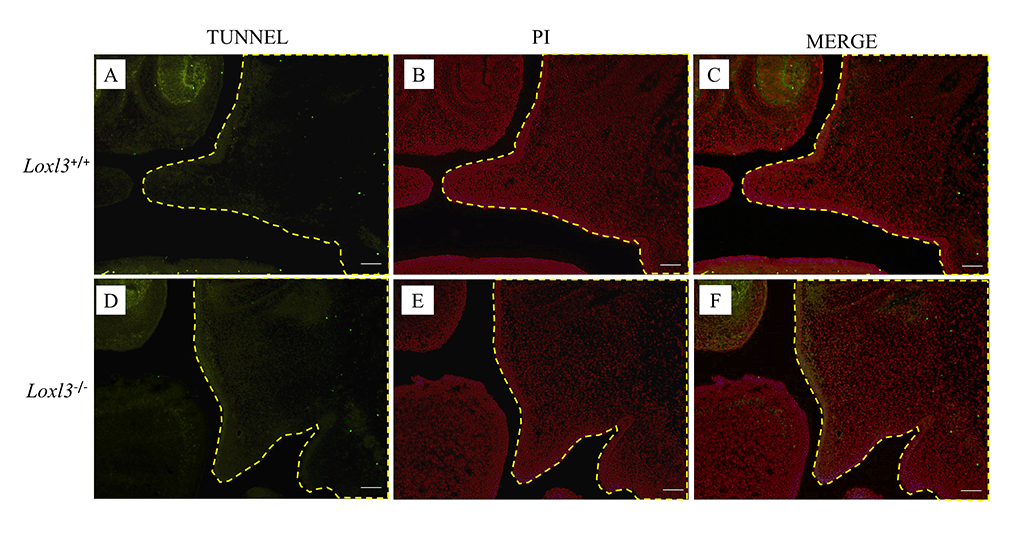


Supplementary Figure S3. Apoptosis in palate shelves of wild-type (A-C) and mutant (D-F) embryos at E14.5. TUNEL-positive (green) cells in mutants had not obvious difference from those in controls. Bar: 100μm.


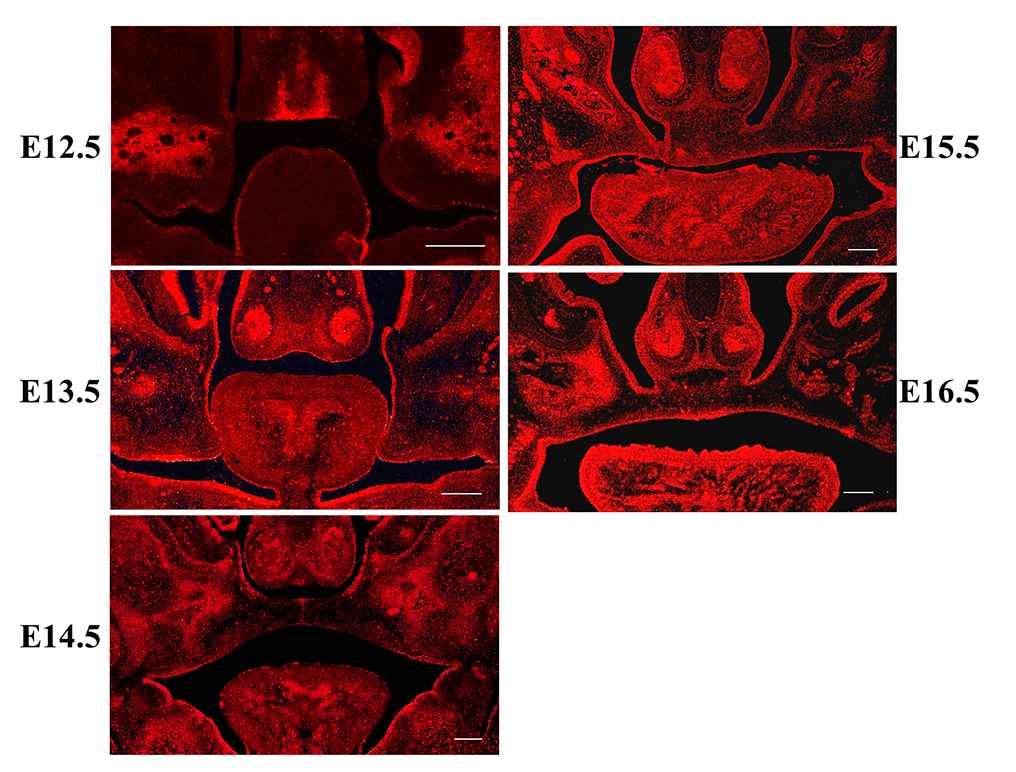


Supplementary Figure S4. Expression of LOXL3 protein in palate at different embryonic periods. Immunofluorescence staining showed that the expression of *Loxl3* was mainly localized to the palate mesenchymal region. Bar: 200μm.
